# A Novel Promazine Derivative Shows High *in vitro* and *in vivo* Antimicrobial Activity Against *Staphylococcus aureus*

**DOI:** 10.3389/fmicb.2020.560798

**Published:** 2020-09-30

**Authors:** Troels Ronco, Nadia S. Jørgensen, Iben Holmer, Sofie Kromann, Ehsan Sheikhsamani, Anders Permin, Søren W. Svenningsen, Jørn B. Christensen, Rikke H. Olsen

**Affiliations:** ^1^Department of Veterinary and Animal Sciences, Faculty of Health and Medical Sciences, University of Copenhagen, Copenhagen, Denmark; ^2^Department of Animal Science, Faculty of Agriculture, Ferdowsi University, Mashhad, Iran; ^3^Unibrains, Virum, Denmark; ^4^Department of Chemistry, Faculty of Science, University of Copenhagen, Copenhagen, Denmark

**Keywords:** phenothiazine derivative, novel antimicrobial compound, topical agent, *Staphylococcus aureus*, skin infections

## Abstract

The emergence of multidrug-resistant bacteria constitutes a significant public health issue worldwide. Consequently, there is an urgent clinical need for novel treatment solutions. It has been shown *in vitro* that phenothiazines can act as adjuvants to antibiotics whereby the minimum inhibitory concentration (MIC) of the antibiotic is decreased. However, phenothiazines do not perform well *in vivo*, most likely because they can permeate the blood-brain (BBB) barrier and cause severe side-effects to the central nervous system. Therefore, the aim of this study was to synthesize a promazine derivate that would not cross the BBB but retain its properties as antimicrobial helper compound. Surprisingly, *in vitro* studies showed that the novel compound, JBC 1847 exhibited highly increased antimicrobial activity against eight Gram-positive pathogens (MIC, 0.5–2 mg/L), whereas a disc diffusion assay indicated that the properties as an adjuvant were lost. JBC 1847 showed significant (*P* < 0.0001) activity against a *Staphylococcus aureus* strain compared with the vehicle, in an *in vivo* wound infection model. However, both *in vitro* and *in silico* analyses showed that JBC 1847 possesses strong affinity for human plasma proteins and an Ames test showed that generally, it is a non-mutagenic compound. Finally, *in silico* predictions suggested that the compound was not prone to pass the BBB and had a suitable permeability to the skin. In conclusion, JBC 1847 is therefore suggested to hold potential as a novel topical agent for the clinical treatment of *S. aureus* skin and soft tissue infections, but pharmacokinetics and pharmacodynamics need to be further investigated.

## Introduction

Across the globe, emerging multidrug-resistant human and veterinary bacterial pathogens constitute a considerable public health issue and cost many lives every year. Among these pathogens, methicillin-resistant *Staphylococcus aureus* (MRSA) and third-generation cephalosporin-resistant *Escherichia coli* are some of the most problematic (Coates et al., 2011; Cassini et al., 2019). Therefore, there is an urgent clinical need for novel treatment solutions but at the same time the number of novel antibiotics introduced to the market has decreased considerably the last decades (Ventola, 2015; Singer et al., 2019). As a result, alternatives to antibiotics have extensively been investigated and previous studies suggest that phenothiazines can be applied as adjuvants to specific antibiotics, thereby increasing the bacterial susceptibility to the antimicrobial agent (Kristiansen et al., 2003, 2006). Phenothiazines constitute a group of neuroactive compounds that can act as a dopamine antagonists and have originally been used as antipsychotics (Snyder et al., 1974). In general, phenothiazines possess low antimicrobial activity compared with commercial antimicrobials and, e.g., the minimum inhibitory concentration (MIC) of promazine against a MRSA strain, has been reported to be 128 mg/L (Nehme et al., 2018). However, when used as helper compounds in combination with specific antibiotics, e.g., beta-lactams, they can cause a synergistic effect by decreasing the MIC of the antibiotic, probably by inhibiting efflux pumps (Kristiansen et al., 2007, 2010). However, it appears to be difficult to apply phenothiazines as an antimicrobial helper compound in clinical practice. The concentration of phenothiazines must be relatively high to obtain the synergistic effects, and studies have revealed that these compounds did not perform well as helper compounds when tested *in vivo*. In some cases, the administrated dose needed to be reduced considerably due to severe side-effects observed in the phenothiazine-treated animals (Stenger et al., 2015, 2017). Since phenothiazines possess lipophilic properties, they can easily cross the blood-brain barrier (BBB) and cause effects on the central nervous systems (CNS) (Seelig et al., 1994). Therefore, we have hypothesized that the toxic side-effects caused by phenothiazines during *in vivo* studies are partly due to effects on the brain. The main purpose of this study was to chemically modify promazine, a compound belonging to the phenothiazine group, so that it would not permeate the BBB but retain its properties as an antimicrobial helper compound. A further purpose was to assess the properties and safety of the novel derivate using *in vitro*, *in vivo*, and *in silico* approaches.

## Materials and Methods

### Synthesis of JBC 1847

A mixture of promazine (1.0 g; 3.3 mmol), 3,7-dimethyloct-6-en-1-yl 4-methylbenzenesulfonate (1.2 g; 3.8 mmol) (synthesized by the procedure by Zarbin et al., 2000) in acetonitrile (25 ml) was heated to + 40°C and kept at + 40°C for 5 days. The acetonitrile was removed *in vacuo*, and diethyl ether (50 ml) was added with stirring to the residue making the product crystallize. The product was isolated by filtration and dried in vacuum to give JBC 1847 a 58% yield (1.2 g). All the chemicals were obtained from Sigma-Aldrich and were used as received.

### Investigating the Antimicrobial Activity *in vitro*

Based on Clinical and Laboratory Standards Institute’s (CLSI’s) guidelines the MIC and the minimum bactericidal concentration (MBC) values for JBC 1847 were determined for 11 human and veterinary clinical isolates (Table 1). Initially, the broth microdilution method was used, and the strains were generally grown aerobically overnight (ON) at 37°C on Mueller-Hinton agar (MHA) (Sigma, Copenhagen, Denmark) supplemented with 5% bovine blood. However, *Cutibacterium acnes* was grown anaerobically at 37°C for approximately 72 h on tryptone soya agar (TSA) (tryptic soy agar, Oxoid, Roskilde, Denmark). Colonies were suspended in 0.9% NaCl to an optical density of 0.5 McFarland standard using a Sensititre^TM^ nephelometer (Thermo Scientific^TM^, Roskilde, Denmark). Subsequently, the suspensions were diluted 100-fold in Mueller-Hinton broth (MHB) and 100 μl of the dilution was transferred to the wells of a sterile 96-well plate containing 100 μl MHB with JBC 1847 resulting in concentrations ranging from 0.02 to 256 mg/L and a final volume of 200 μl. The inoculum of all wells including positive controls containing only MHB, was therefore 1.5 × 10^5^ colony forming units (CFU) whereas the negative controls contained only MHB. All 96-well plates were grown aerobically as previously described except *C. acnes* that was incubated anaerobically as previously described in brain heart infusion (BHI; Sigma, Copenhagen, Denmark) media with 1% L-cysteine (Blaskovich et al., 2019). The concentration in wells with no visible bacterial growth was defined as the MIC. The MBC for all isolates was determined by culturing 10 μl from the MIC wells and all other wells with no visible growth, on TSA aerobically as previously described. The MBC for *C. acnes* was determined on BHI agar with 1% L-cystein incubated as previously described. MBC was defined as the lowest concentration of JBC 1847 that reduced the CFU of the original inoculum (1.5 × 10^5^ CFU) by ≥ 99.9%. Both MIC and MBC values were determined in biological duplicates except for *C. acnes* and *Staphylococcus epidermidis* where only technical duplicates were used.

**TABLE 1:** Broth microdilution results for JBC 1847.

Species	Strain	Origin	MIC (mg/L)	MBC (mg/L)
*Staphylococcus aureus*	USA300 JE2	Human clinical isolate	0.5	0.5
*S. aureus*	ATCC BAA-1556	Human clinical isolate	0.5	0.5
*S. aureus*	CC398 (in house strain)	Veterinary clinical isolate	0.5	0.5
*Staphylococcus epidermidis*	RP62a	Human clinical isolate	0.5	ND
*S. epidermidis*	105 (in house strain)	Human clinical isolate	0.5	ND
*Cutibacterium acnes*	P10140156 (in house strain)	Human clinical isolate	0.5–1	0.5–1
*Enterococcus faecalis*	JEO72G7B	Veterinary clinical isolate	1	1
*Enterococcus faecium*	ATCC 700221	Human clinical isolate	2	2–4
*Proteus vulgaris*	JEO1	Veterinary clinical isolate	32	32
*Escherichia coli*	E2 (in house strain)	Human clinical isolate	8	8
*E. coli*	APEC O2 (in house strain)	Veterinary clinical isolate	16	16

*The table shows minimal inhibitory concentrations (MIC) and minimal bacterial concentrations (MBC) of JBC1847 determined for 11 human or veterinary clinical isolates. ND, not determined.*

In addition, whether serum proteins affected the activity of JBC 1847 against three *S. aureus* strains (USA300 JE2, ATCC BAA-1556 and CC398 in house strain) was investigated. Broth microdilution assays analog to previously described, with a 20% human serum (Sigma-Aldrich, Brøndby, Denmark) concentration were carried for three *S. aureus* strains (Table 1). MIC and MBC values were determined as previously described.

To determine if eight different antibiotics from six classes had an impact on the activity of JBC 1847, agar dilution, and disc diffusion assays based on CLSI’s guidelines were performed with two *S. aureus* strains (USA300 JE2 and ATCC-BAA). Initially, MHA plates with JBC 1847 concentrations ranging from 0.5 to 8 mg/L were made and bacterial inoculum was prepared as previously described, was added to each plate. After incubation as previously described, the MIC was determined as the lowest concentration that resulted in inhibition of bacterial growth. The MIC values for the two *S. aureus* strains were both determined to 4 mg/L and therefore, TSA plates with a JBC 1847 concentration of 2 mg/L were prepared to ensure the strains were able to grow. One dish of each type of antibiotic was added to a single TSA plate with JBC 1847 and a control plate containing only TSA and inoculated as previously described. Two plates containing only 2 mg/L JBC 1847 and bacterial inoculum for each strain served as a positive control. After incubation, clearing zones were measured and compared between control and JBC 1847 plates.

### Induced JBC 1847 Tolerance

To study if resistance toward JBC 1847 is easily developed, *S. aureus* USA300 was continuously exposed to increasing concentrations of JBC 1847 during a period of 23 days in an assay inspired by a previous study (Wassmann et al., 2018). This assay was carried out in technical duplicates as described for the MIC assay, except that concentrations of JBC 1847 varied. Initially, USA300 colonies were used to prepare a McFarland solution with an optical density of 0.5. The McFarland solution was diluted in MHB and used as inoculum in wells with a final concentration of 0.25, 0.5, and 0.75 mg/L JBC 1847 and a final volume of 200 μl. The 96-well plate was grown aerobically ON at 37°C, and the next day, the highest concentration where growth was observed was in the 0.5 mg/L well. Therefore, bacterial culture from this well was diluted 1:100 and used as inoculum for wells with a final concentration of 0.5, 0.75, and 1.0 mg/L JBC 1847. This cycle was repeated during a period of 23 days and if no increase in resistance was observed the strain was grown in the same concentrations as the previous day. However, in the last 8 days, the assay was performed in 20 ml test tubes in a volume of 2 ml. As for the 96 wells, pure MHB served as negative control, whereas inoculum in pure MHB served as positive control. Approximately every 2nd day during the 23 days period, 10 ml of the daily inoculum was transferred to MH blood agar plates and cultivated as previously described to ensure no contamination was present. Simultaneously, an identical assay was performed using fusidic acid natrium salt (Sigma). Here, the starter concentration was 0.03 mg/L corresponding to the MIC value for USA300, determined using the broth microdilution method as previously described. The daily concentration was first increased by 0.02 mg/L, but later on, during the period of 23 days, the concentrations were increased by values ranging from 0.250 to 1 mg/L.

### Growth and Viability Assays

Growth and viability assays were performed according to a previously published method (Thorsing et al., 2013), with some modifications. In general, the assay was conducted over a 24 h period in a 37°C heating bath with shaking, using *S. aureus* USA300 JE2 as test strain. The strain was exposed to sub- and supra-MIC of JBC 1847, and for each concentration, samples were collected at eight different timepoints during the 24 h period. To determine the MIC for the test strain, a single colony incubated on MHA with blood at 37°C for 20–22 h was inoculated into BHI broth and further incubated at 37°C with shaking for 20–22 h. The culture was diluted 1:100 in BHI broth and grown to early exponential phase (OD_600_ 0.20). The culture was divided into four flasks each with a volume of 100 ml BHI broth and a JBC 1847 concentration of 2, 4, 8, or 16 mg/L. The flasks were grown in 37°C with shaking for 20–22 h, and the MIC was visually determined to 4 mg/L. To test the viability of USA300 JE2 when exposed to JBC 1847, an ON culture was prepared and diluted as previously described and 4 ml was subsequently added to 400 ml BHI broth. This culture was grown to OD_600_ 0.20 where sample zero was collected. Subsequently, the culture was divided into four flasks whereof one served as untreated control and the remaining three had a concentration of 2, 4, and 16 mg/L. Flasks were incubated at 37°C with shaking, and samples were collected when adding JBC 1847 and 1, 2, 3, 4, 5, 8, and 24 h after. 100 μl samples were serially diluted, spread on MHA plates, and incubated at 37°C for 20–24 h followed by successive counting.

### Transmission Electron Microscopy

To elucidate the interaction between JBC 1847 and MRSA USA300, transmission electron microscopy (TEM) was carried out. Bacterial cultures were inoculated in 10 ml MHB and cultivated at 37°C for 5 h, with shaking, until mid-exponential growth phase (OD_550_ 5.5). Once mid-exponential phase was reached, the cells were treated according to the sub- and supra-MIC (0.5 × MIC, MIC, 2 × MIC, and 4 × MIC) of JBC1847 at 37°C for 1 h. An untreated control was also prepared. The five samples were placed on ice until being prepared for TEM. This included a fixation step in 2% glutaraldehyde in 0.15 M sodium phosphate buffer (pH 7.2). Subsequently, the fixed cells were washed three times and postfixed with 0.2% OsO_4_ (osmium tetroxide) in H_2_O/0.15 M sodium phosphate buffer (pH 7.2) for 1 h. The samples were then dehydrated with graded acetone, and then embedded in epoxy resin. Ultrathin sections of the samples were prepared using Formvar-coated copper grids, which were stained using 3% uranyl acetate. The samples were placed in a Philips CM 100 Transmission EM (Philips, Eindhoven, Netherlands) and exposed to 120 keV electron energy.

### Assessment of the Efficacy of JBC 1847 *in vivo*

To study the effect of topical treatment with JBC 1847 against MRSA strain 43484, a mouse skin infection model was established. Initially, female mice (18–22 g) were orally treated with 45 μl nurofen (20 mg ibuprofen/ml) and anaesthetized with 0.15 ml s.c. of Zoletil mix. The fur was removed from an area of 6 cm^2^ and a part of epidermis was subsequently scraped off to obtain a 1 cm^2^ superficial skin lesion. The mice were inoculated with 10 μl of a saline solution containing approximately 10^7^ CFU. The day after inoculation, topical treatment with 50 μl test compound was initiated and performed twice daily for a 3-day period. Fucidic acid was included as a positive control, and vehicle treatment was included as a negative control. Through the entire study, the behavior and clinical signs of the mice were carefully observed. Mice were killed the day after last treatment, and skin biopsies from the wounded area were collected. The skin samples were homogenized using a Dispomixer and serially diluted in saline/Triton X. To determine the bacterial concentration, 20 μl spots were transferred to MRSA Brilliance agar plates (Oxoid, Thermo Scientific, Roskilde, Denmark) and incubated 20–48 h at 35°C.

### Pharmacological Predictions *in silico*

Online tools and software packages were applied to predict the pharmacodynamics and pharmacokinetics of JBC 1847. The following three tools were used: VEGA-QSAR, v1.1.5 (Benfenati et al., 2013), SwissADME (Daina et al., 2017), and PreADMET (PreADMET, 2019) together with the two SMILES formulas.

JBC 1847: (CC(CCC = C(C)C)CC[N + ](C)(C)CCCN1C2=C (SC3=C1C=CC=C3)C=CC=C2).

Promazine: (CN(C)CCCN1C2 = CC = CC =C2SC3=CC=C C=C31).

The formulas were converted to MOLfiles using the online server Cheminfo.org, 2019.

### Ames Test

To investigate if JBC 1847 exhibited mutagenic properties, Ames mutagenicity test was performed. In this reverse mutation assay, five bacterial tester strains recommended by The Organization for Economic Cooperation and Development (OECD), were exposed to JBC 1847 in presence and absence of a metabolic activation system (S9) obtained from rat liver extract (Maron and Ames, 1983; Levy et al., 2019). The characteristics of the four *Salmonella typhimurium* mutants (TA98, TA100, TA1535, TA1537) and a single *Escherichia coli* mutant [wp2 (pKM101)] have been described by Mortelmans and Zeiger (2000) and Levy et al. (2019). Prior to testing, the genotypes of all strains were confirmed as previously described (Mortelmans and Zeiger, 2000), and JBC 1847 was tested in various concentrations (1.5, 4, 10, 25, 64, 160, 400, and 1,000 μg/well) whereas DMSO served as negative control. As positive controls, five mutagenic compounds were applied in various concentrations (Supplementary Material). The Ames test was performed as described in Supplementary Material where acceptance criteria for the test results can also be found. To evaluate the test results, the mutagenicity ratio (MR) was calculated and interpreted as in previous studies (Pah and Lakhani, 2012; Wang et al., 2018). The MR was calculated as MR = *x*/*x*_n_. Here, *x* was the number of reverse mutated colonies exposed to JCB 1847 whereas *x*_n_ was the number of reverse mutated colonies in the negative controls. A MR of ≥ 2 was interpreted as positive regarding mutagenicity.

### Statistical Analyses

Data were statically analyzed and visualized using RStudio v3.6.3 (R Core Team, 2020) and Microsoft excel. In general, the values reflecting the bacterial concentration (CFU/ml) were transformed into a logarithmic scale. In the growth and viability assay, the mean numbers of the bacterial concentrations from the triplicates were plotted against time and standard deviations shown as error bars. To investigate differences in bacterial concentration between treatment groups in the *in vivo* assay, multiple group comparisons were performed using ANOVA and *P* < 0.05 considered significant. The data was visualized in a standard box plot.

## Results

### Synthesis of JBC 1847

As mentioned in the “INTRODUCTION,” phenothiazines can easily cross the BBB and possibly cause negative effects on the CNS (Seelig et al., 1994). We hypothesized that adding an alkyl group to the tertiary amine of promazine to form a quaternary ammonium ion would prevent a compound from penetrating the BBB. The choice of a citronnelyl group (systematic name: 3,7-dimethyloct-6-en-1-yl) as a tail, was based on an idea that the structure of the tail (which is a monoterpene) might be recognized as a potential carbon and energy source (Marmulla and Harder, 2014) and may help the rest of the molecule to enter the bacteria.

The yield of JBC 1847 was 1.2 g (58%). HRMS (ESI): 423.2879 (M^+^, calcd. for C_2__7_H_3__9_N_2_S^+^: 423.28). ^1^H-NMR (500 MHz, CDCl_3_) *δ*: 7.77 (d, 2H, *J* = 10 Hz), 7.22–7.17 (m, 4H), 7.14 (d, 2 H, *J* = 10 Hz), 7.00–6.96 (m, 2H), 6.92–6.90 (m, 2H), 5.04 (s, 1H), 4.09–4.03 (m, 2H), 3.64–3.56 (m, 2H), 3.20–3.06 (m, 8H), 2.37 (s, 3H), 2.28–2.19 (m, 2H), 1.70 (s, 3H), 1.60 (s, 3H), 1.46–1.10 (m, 2H), 1.30–1.10 (m, 3H), 1.00–0.91 (m, 2H), and 0.69 (d, 3 H, *J* = 10 Hz). ^13^C-NMR (126 MHz, CDCl_3_) δ: 144.65, 143.97, 139.08, 131.76, 129.80, 128.59, 127.81, 126.02, 125.91, 123.89, 123.27, 116.30, 62.52, 60.87, 51.56, 43.76, 36.71, 30.22, 28.96, 25.74, 25.22, 21.31, 20.07, 18.76, and 17.75.

Elemental analysis: calcd. for C_3__4_H_4__6_N_2_O_3_S_2_: C, 68.65%; H, 7.79%; and N, 4.71%. Found: C, 68.37%; H, 7.56%; and N, 4.67%.

**FIGURE 1:**
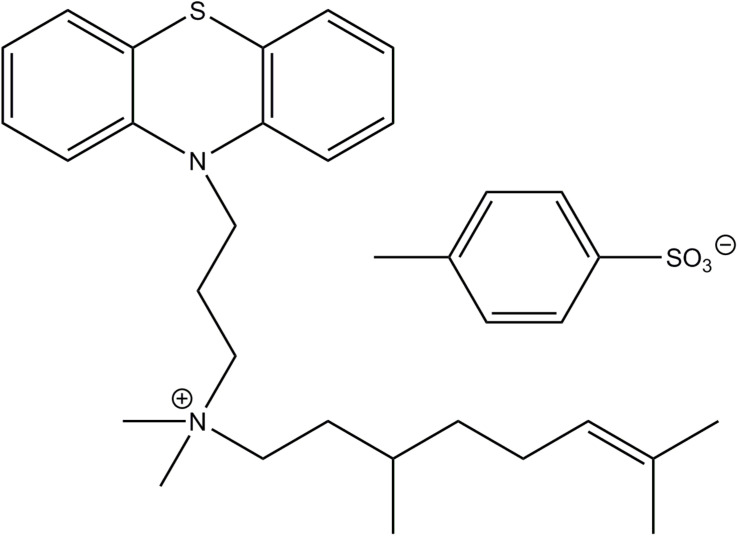
The structure of JBC 1847 and its anion.

The structure of JBC 1847 and its anion is shown in Figure 1.

### Antimicrobial Activity *in vitro*

JBC 1847 was tested for activity against eight Gram-positive and three Gram-negative pathogenic bacteria using the broth microdilution method. The MIC values ranged from 0.5 to 2 mg/L for Gram-positive and from 8 to 32 mg/L for Gram-negative bacteria (Table 1). In all cases except for *E. faecium* ATCC 700221, the MIC values corresponded to the MBC values. When JBC 1847 was tested against three clinical MRSA isolates (USA300 JE2, ATCC BAA-1556 and CC398 in house strain) in a broth microdilution assay with a 20% human serum concentration, the MIC and MBC value were found to be 64 mg/L for all three strains. This is a 128-fold increase (from 0.5 to 64 mg/L) in both the MIC and the MBC values for all three strains, compared with the assay without human serum (Table 1).

In addition, whether JBC 1847 had an impact on the activity of eight antimicrobial compounds belonging to six different classes against two clinical MRSA strains was investigated. No remarkable synergistic or antagonistic effects were observed, and for both test strains, the change in diameter of the clearing zone ranged from −1.7 to 12% between control and test plates (Tables 2, 3). However, when strain ATCC-BAA 1556 was tested in the presence of tetracycline, a 25% change in diameter between control and test plates was observed (Table 2).

**TABLE 2:** JBC 1847 activity against *Staphylococcus aureus* ATCC-BAA 1556 in presence of eight antibiotics.

Antibiotic (μg)	2 mg/L JBC 1847 (mm)	Control plates (mm)	Change (%)
Ampicillin (10)	30–32	30	3.3
Oxacillin (1)	20	18	11.1
Erythromycin (15)	17	17	0
Enrofloxacin (5)	18	17	5.9
Vancomycin (30)	12	12	0
Tetracycline (30)	10	8	25
Gentamicin (10)	22	21	4.8
Ciprofloxacin (5)	12	10	12

*The table shows clearing zones (mm) observed when eight antibiotics were tested against S. aureus ATCC-BAA 1556 in absence (control) and presence of JBC 1847 (2 mg/L). The change (%) indicates the difference in clearing zones.*

**TABLE 3:** JBC 1847 activity against *Staphylococcus aureus* USA300 in presence of eight antibiotics.

Antibiotic (μg)	2 mg/L JBC 1847 (mm)	Control plates (mm)	Change (%)
Ampicillin (10)	31–33	28–30	10.3
Oxacillin (1)	18–20	18–20	0
Erythromycin (15)	27–30	28–30	- 1.7
Enrofloxacin (5)	18	17	5.9
Vancomycin (30)	10	10	0
Tetracycline (30)	27	27	0
Gentamicin (10)	21	20	5
Ciprofloxacin (5)	10–12	10	10

*The table shows clearing zones (mm) observed when eight antibiotics were tested against S. aureus USA300 JE2 in absence (control) and presence of JBC 1847 (2 mg/L). The change (%) indicates the difference in clearing zones.*

### Induced JBC 1847 Tolerance

The results showed that the increase in MIC for fusidic acid (233-fold) was remarkably high compared with JBC 1847 (7-fold) when tested against MRSA USA300 during the 23 days (Table 4). In addition, the highest MIC (3.5 mg/L) for JBC 1847 was reached at day 4, and hereafter, these MIC values ranged from 1 to 2.5 mg/L. In contrast, the MIC value for fusidic acid was continuously increasing and peaked with 7 mg/L on the final day (Table 4).

**TABLE 4:** Tolerance assay.

Compound	MIC (mg/L)	MIC (mg/L)	Increase (fold)
	Day 1	Day 23	
JBC 1847	0.5	3.5	7
Fusidic acid	0.03	7	233

*The table shows minimal inhibitory concentrations (MIC) for JBC 1847 and fusidic acid against Staphylococcus aureus USA300 JE2 at days 1 and 23 during a 23-day test period. Additionally, the increase (fold) in MIC is shown for both compounds.*

### Growth and Viability Assays

**FIGURE 2:**
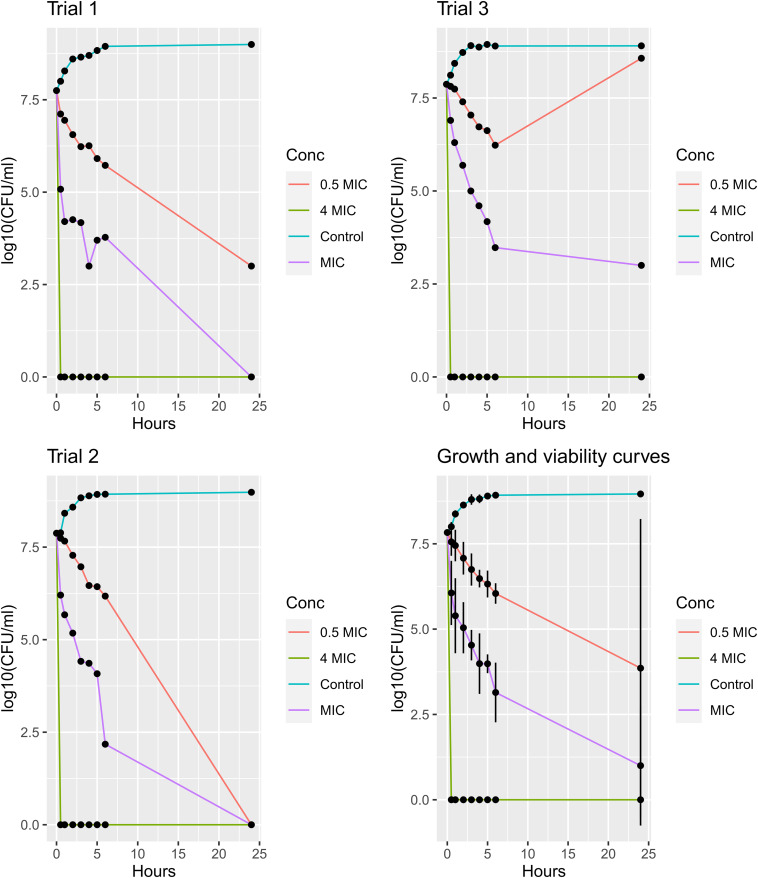
Growth and viability assay. The plots show growth and viability curves for *Staphylococcus aureus* USA300 JE2 exposed to JBC 1847 in three concentrations (0.5 × MIC, MIC, and 4 × MIC) together with an untreated control for 24 h. The assays were performed in triplicates (trials 1, 2, and 3), and the mean numbers of the bacterial concentrations [log10(CFU/ml)] from all triplicates including standard deviations presented as error bars, are shown in the plot at the bottom right.

To study the correlation between the concentration of JBC 1847 and viability of *S. aureus* USA300 JE2 an assay lasting for 24 h was carried out in triplicates, as seen in Figure 2. When the concentration of JCB 1847 was 0.5 × MIC, a prolonged phase was observed, and after 24 h, the bacterial concentration reached approximately 10^3^ and 0 CFU/ml in trials 1 and 2, respectively. However, in trial 3, the concentration started to increase after 6 h and reached the same level as the control curve after 24 h. At the MIC concentration, the bacterial concentration was constantly decreasing, and at trials 1 and 2, the bacterial concentration reached 0 CFU/ml at 24 h. However, in trial 3, the bacterial count was measured to approximately 10^3^ CFU/ml after 24 h. At a concentration of 4 × MIC, no viable bacterial cells were measured after 30 min in all three replicates (Figure 2).

### Transmission Electron Microscopy

**FIGURE 3:**
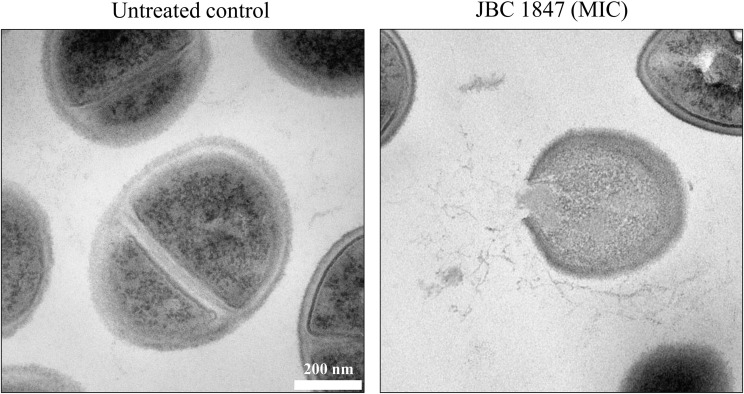
Transmission electron microscopy. The two transmission electron microscopy (TEM) photos show *Staphylococcus aureus* USA300 JE2 untreated and treated with JBC 1847 in MIC.

To obtain an overview of how JBC 1847 interacts with bacteria, TEM was carried out. Compared with the untreated control, the bacterial cells were clearly lysed and the plasma membrane destroyed, when exposed to JCB 1847 in a concentration corresponding to the MIC (Figure 3).

### Efficacy of JBC 1847 in an *in vivo* Wound Model

**FIGURE 4:**
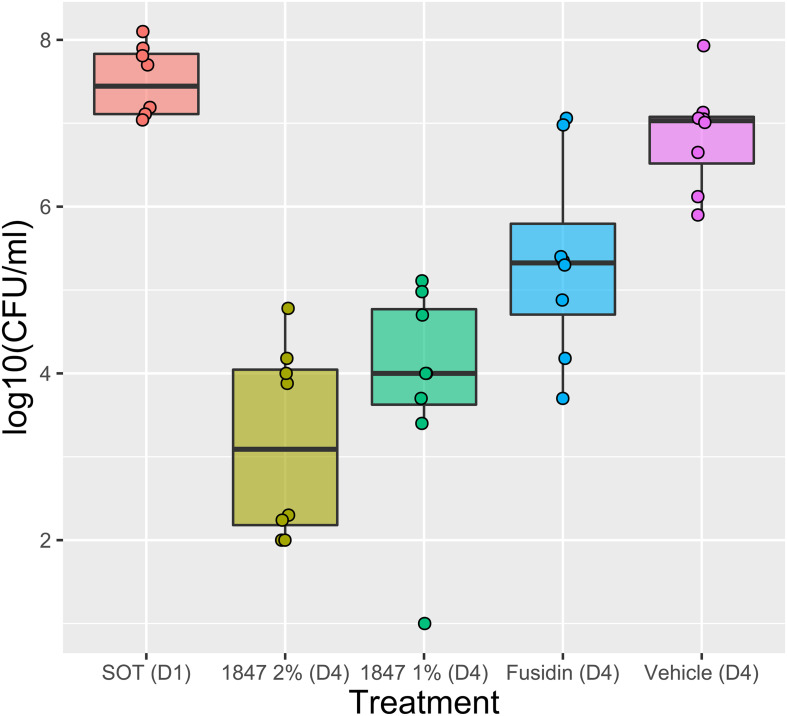
Efficacy of JBC 1847 against *Staphylococcus aureus in vivo*. The box plot shows bacterial counts [log10(CFU/ml)] from a 4-day wound infection experiment. The sample median is shown within the boxes and the first and third quartile is shown as the upper and lower edge of the boxes, respectively. At day 4 (D4), the bacterial counts were significantly decreased (*P* < 0.0001) when wounds were treated with 1 and 2% JBC 1847 (1847), compared with the vehicle treatment. Treatment with 2% Fusidin (fusidic acid) did also result in a significant reduction (*P* < 0.05) in bacterial counts. SOT (D1) is the start of treatment on day 1 (D1).

To study the efficacy of JBC 1847 against MRSA in skin lesions, a murine wound model was performed. The colony count in the inoculum was determined to 8.8 log10 CFU/ml, corresponding to 6.8 log10 CFU/mouse. During the test period, no mice showed any clinical signs of infection or discomfort in general. The mean number of bacterial concentrations of the vehicle (negative control) was determined to 6.86 log10 CFU. Compared with the vehicle, solutions of 1 and 2% JBC 1847 reduced the bacterial counts significantly (*P* < 0.0001) with 3.0 and 3.7 log10 CFU, respectively (Figure 4). Treatment with 2% fusidic acid resulted in a 1.5-log10-CFU reduction of the bacterial count compared with the vehicle (*P* < 0.05) (Figure 4). Notably, when counting colonies in spots of 10-fold dilutions of the skin samples, a carry-over effect was observed.

### Pharmacological Predictions of JBC 1847 *in silico*

To assess the pharmacological properties of JBC 1847 compared with its mother compound promazine, various pharmacodynamic and pharmacokinetic analyses were performed using different Web programs. The results predicted that JBC 1847 will not cross the BBB and possesses a strong affinity for plasma proteins (Table 5). In contrast, promazine was predicted to be able to cross the BBB and had a weak affinity for plasma proteins. Both compounds were found to be less prone to permeate the skin since both had a relatively low Log *K*_p_ [the more negative the log *K*_p_, the less skin permeant the molecule (Edwards and Langer, 1994)]. In addition, Table 5 shows that identical tests resulted in different outputs according to which programs were used; e.g., VEGA-QSAR predicted JBC 1847 to be non-mutagenic in the Ames test whereas PreADMET predicted it to be a mutagenic compound. In comparison, promazine was found to be non-mutagenic in both cases. Moreover, JBC 1847 was found to be a non-carcinogen except in the PreADMET rat model, whereas promazine was found to be a carcinogen in all cases. Both JBC 1847 and promazine were identified as cytochrome P450 2D6 (CYP2D6) enzyme inhibitors with a medium risk of being hERG channel inhibitors. Finally, SwissADME predicted a low gastrointestinal (GI) absorption of JBC 1847, whereas PreADMET predicted a middle absorption with Caco-2 cell lines. Caco-2 cell lines are used to predict human intestinal absorption (Breemen and Li, 2005).

**TABLE 5:** Pharmacological predictions *in silico.*

Program	Test	JBC 1847	Promazine
VEGA-QSAR	Ames test, CONSENSUS model 1.0.3.	Non-mutagenic	Non-mutagenic
VEGA-QSAR	Carcinogenicity model (CAESAR) 2.1.9.	Non-carcinogen (OBS reliability)	Carcinogen
SwissADME	CYP2D6 inhibitor	Yes	Yes
SwissADME	Log *K*_p_ (skin permeation)	−3.05 cm/s	−4.80 cm/s
SwissADME	GI absorption	Low	High
SwissADME	Blood-brain barrier permeability	No	Yes
PreADMET	hERG inhibition	Medium risk	Medium risk
PreADMET	Ames test	Mutagen	Non-mutagen
PreADMET	Carcino_Mouse	Negative	Positive
PreADMET	Carcino_Rat	Positive	Positive
PreADMET	Caco-2	23.0603	23.0271
PreADMET	CYP2D6 inhibitor	Yes	No
PreADMET	Plasma protein binding	93.43% (strong)	81.82% (weak)

*The table shows important pharmacological parameters for JBC 1847 and promazine determined by threes software programs. hERG, human ether-a-go-go-related gene.*

### Ames Test

The Ames test met all criteria for being a valid test (Supplementary Material), and the genotypes of all strains were confirmed. The mean number of reverse mutated colonies is presented in Table 6. In general, JBC 1847 was found to be a non-mutagenic compound with MR values of < 2. However, the MR for *S. typhimurium* TA1537 was above 2 at a concentration of 4 μg/well in the presence and absence of the metabolic activation system S9 (Hubbard et al., 1985; Table 6).

**TABLE 6:** Ames test results.

Concentration (μg/well)	TA98		TA100		TA1535		TA1537		*Escherichia coli*	
	+ S9	−S9	+ S9	−S9	+ S9	−S9	+ S9	−S9	+ S9	−S9
1.5	11.00 ± 2.65	13.00 ± 2.00	21.33 ± 2.08	24.33 ± 3.21	24.33 ± 3.21	24.33 ± 3.21	2.00 ± 2.65	5.00 ± 1.00	51.33 ± 12.86	33.00 ± 2.65
4	11.33 ± 3.79	9.00 ± 2.00	19.00 ± 5.29	26.67 ± 2.08	26.67 ± 2.08	26.67 ± 2.08	4.33^a^ ± 0.58	7.67^a^ ± 2.08	40.67 ± 9.07	39.33 ± 5.69
10	13.67 ± 4.04	9.67 ± 3.51	13.67 ± 1.53	18.00 ± 5.57	18.00 ± 5.57	18.00 ± 5.57	0.33 ± 0.58	NC	40.67 ± 13.32	17.33 ± 7.09
25	NC	0.0	NC	0.0	NC	0.0	NC	NC	9.67 ± 2.08	NC
64	0.0	0.0	0.0	0.0	0.0	0.0	0.0	0.0	0.0	0.0
Negative control	9.50 ± 3.27	7.33 ± 2.25	22.67 ± 3.88	21.33 ± 6.12	1.5 ± 1.76	1.17 ± 1.17	1.83 ± 1.60	2.67 ± 1.21	39.33 ± 8.36	35.00 ± 3.90
Positive control	332.0 ± 36.66	268.00 ± 28.84	240.0 ± 49.15	176.0 ± 46.13	34.00 ± 5.20	111.0 ± 9.54	60.00 ± 5.00	54.67 ± 16.26	161.33 ± 18.90	206.67 ± 16.65

*The table shows mean numbers of reverse mutant colonies and standard deviations, for each of the five tester strains [Salmonella typhimurium TA98, TA100, TA1535, TA1537, and E. coli wp2 (pKM101)] when exposed to various concentrations (Conc.) of JBC 1847. In addition, the results of the negative and positive controls are shown. NC, not counted. ^a^Mutagenicity ratio (MR) of ≥ 2 and NC.*

## Discussion

The main purpose of this study was to synthesize a less-toxic derivative of promethazine that would not cross the BBB but retain its properties as an antimicrobial helper compound (Shibl et al., 1984). This resulted in JBC 1847, and according to *in silico* analyses, this compound was not permeable to the BBB whereby it will act less toxic to the CNS compared with its mother compound, promazine. JBC 1847 unexpectedly exhibited highly increased antimicrobial activity compared with promazine but seemed to have lost its properties at helper compound. A previous study has determined the MIC for promazine to be 128 mg/L for both MRSA and *E. coli* strains using the broth dilution method (Nehme et al., 2018). In contrast, the MIC for JBC 1847 was 0.5 mg/L when tested against three MRSA strains and 8/16 mg/L when tested against two *E. coli* strains (Table 1). Generally, JCB 1847 exhibited most efficient activity against Gram-positive bacteria (MIC range, 0.5–2 mg/L) compared with the Gram-negative bacteria (MIC range, 8–32 mg/L). In almost all cases the MIC corresponded to the MBC values except for *E. faecium* ATCC 700221. This indicates that JBC 1847 can be considered a bactericidal compound since MIC and MBC do not vary 4-fold from each other (Rhee and Gardiner, 2004). Furthermore, a disc diffusion assay strongly indicated that JBC 1847 had lost its property to act as an antimicrobial adjuvant. No remarkable synergistic or antagonistic effects were observed when tested against two MRSA strains in the presence of eight well-known antibiotics from six different classes (Tables 2, 3). As previously mentioned, several compounds from the phenothiazine group have previously been reported to act as antimicrobial adjuvants (Kristiansen et al., 2003, 2006, 2007, 2010). JCB 1847 was tested for synergistic properties in a 0.5 × MIC concentration (2 mg/L). Usually, the concentration of phenothiazines have been 5–10-fold higher (10–20 mg/L) than in the present study when tested as a helper compound to oxacillin against MRSA (Kristiansen et al., 2003, 2006). It could be speculated if JBC 1847 could still work efficiently as helper compound if the concentration is raised. To further analyze its antimicrobial properties, a MRSA strain was exposed to JBC 1847 in a growth and viability assay, and generally, concentration-dependent kinetics was observed. The control curves for untreated bacteria and the viability curves for the 4 × MIC concentration turned out as expected and exposure to four MIC resulted in a total decline in the bacterial concentration within 30 min (Figure 2). At a concentration of 0.5 × MIC, a prolonged lag phase as a result of adaption to an unfavorable growth environment could have been expected (Møller et al., 2016), followed by an exponential growth phase. Only one of the replicates (trial 3) reached an exponential phase, and no real lag phase was observed for any of the triplicates at this concentration. At the MIC, a bactericidal effect was observed for two replicates (trials 1 and 2). This was expected since JBC 1847 had previously exhibited bactericidal properties (Rhee and Gardiner, 2004) in the broth microdilution assay (MIC corresponded to MBC). However, in the third replicate (trial 3), the bacterial concentration did not reach zero within 24 h. It is difficult to conclude precisely on these varying results between the replicates for the MIC. It could be suggested that JBC 1847 was not heated nor vortexed sufficiently to be a homogeneous liquid whereby a decreased concentration was obtained at trial 3 compared with trials 1 and 2. During the assay, contamination could have occurred and hence, blood agar could have been applied when determining the bacterial count to ensure via morphology that the culture had remained uncontaminated during the experiment.

In addition, how easily a MRSA strain would develop resistance to JBC 1847 compared with fusidic acid was investigated in a 23-day period. During this period, the MIC value for fusidic acid was increased 233-fold compared with 7-fold for JBC 1847 (Table 4). This suggests that it is more difficult for *S. aureus* to develop resistance against JBC 1847 compared with fusidic acid. Previously, fusidic acid-resistant *S. aureus* isolates have been widely reported to constitute an emerging issue in clinical settings (O’Neill et al., 2007; Castanheira et al., 2010; Yu et al., 2015) and highly elevated MIC values of ≥ 512 μg/ml have been observed (Castanheira et al., 2010).

When the JBC 1847 activity against three MRSA strains (Table 1) was investigated in the presence of human serum proteins, the ordinary MIC and MBC values were remarkably increased by 128-fold (from 0.5 to 64 mg/L) for all three strains. This strongly indicates that JBC 1847 binds to human serum proteins which corresponded to the *in silico* analysis that also predicted a strong plasma protein binding (PPB) properties for JCB 1847 (Table 5). This further indicates that JBC 1847 is not suitable for the clinical treatment of sepsis where the free fraction of the antimicrobial compound is required to be high. An elevated degree of PPB will most likely result in a low free fraction of the pharmacologically active compound (Zeitlinger et al., 2011). However, well-known antibiotics such as fusidic acid (PPB, approximately 98%) (Zeitlinger et al., 2011) and ceftriaxone (PPB, 90–95%) (Dutta et al., 2001) exhibit a high degree of PPB. Fusidic acid is widely used as a topical agent for the clinical treatment of skin and wound infections caused by *S. aureus* (Kraus and Burnstead, 2011). Considering the efficacy of an antimicrobial agent for dermatological treatment, the degree of PPB is not specifically important. Here, a sufficient skin permeability coefficient (*K*_p_) value is more essential to ensure a stable concentration of the agent in the treated area instead of spreading it systemically. The skin permeability coefficient for fusidic acid was predicted *in silico* to −5.54 cm/s using SwissADME (data not shown) which is not far from JBC 1847 which had a coefficient of −3.05 cm/s (Table 5). In addition, the efficacy of the compound was tested against a MRSA strain in an *in vivo* wound model. JBC 1847 reduced the bacterial concentration in the wounds significantly (*P* < 0.0001) compared with the vehicle treatment and twice as much compared with fusidic acid (Figure 4). Notably, a carry-over effect (Lounis et al., 2008) was observed which indicates that a high concentration of active compound was still present in the skin biopsies at the time of sampling. Therefore, the number of viable bacteria detected on the agar plates during determination of the bacterial counts may have been underestimated. To avoid a carry-over effect, an extra-centrifugation step followed by homogenization and rediluting can be included. Moreover, the experiment lasted for only 4 days, and an extended time period would have provided a more solid data.

To assess the mutagenicity of JBC 1847, Ames test was performed. In general, JBC 1847 has been shown as a nonmutagenic compound with a MR of <2. However, in two cases, when *S. typhimurium* TA1537 was tested in the absence and presence of a metabolic activation system (S9), the MR was found to be ≥ 2 (Table 6). This could indicate a slight potential for mutagenicity (Pah and Lakhani, 2012; Wang et al., 2018) which was also predicted *in silico* (Table 5). In the present study, S9 was derived from rat liver, and notably, previous studies have shown that the outcome of the Ames test may vary according to whether S9 is prepared from rat or human liver extract (Hakura et al., 2005). The S9 system is applied because many xenobiotics are only mutagenic after being metabolized, e.g., by cytochrome P450 enzymes (Hubbard et al., 1985). In fact, many liver and cutaneous enzymes involved in metabolism are the same. However, the frequency of metabolic activity in epidermis is much lower compared with that in the liver. Studies have shown that the phase I metabolic enzyme CYP2D6 is found both in the human liver and skin (Oesch et al., 2007; Zhang et al., 2009). The *in silico* analyses predicted JBC 1847 to be a CYP2D6 inhibitor (Table 5). Altogether, this means that the pharmacokinetics and pharmacodynamics of JBC 1847 needs to be studied more in-depth including the assessment of a human skin model (Zhang et al., 2009).

## Conclusion

The emergence of antimicrobial resistance is an increasing global threat to the public health and therefore, there is an urgent clinical need for novel antimicrobial compounds. Here, we present a novel promazine derivative, JBC 1847, which exhibited a remarkable increased antimicrobial activity. *In vitro* studies showed efficient microbial activity specifically against Gram-positive bacteria and JBC 1847 did also perform well in an MRSA *in vivo* wound model. In addition, an MRSA strain developed a remarkable low degree of resistance toward JCB 1847 compared with fusidic acid. The activity of JBC 1847 against two MRSA strains was not considerably affected by the presence of eight antibiotics belonging to six different classes, which indicated that the properties of acting as an adjuvant have been lost. *In silico* predictions showed that JBC 1847 will not pass through the BBB and possess a low skin permeability potential. Both *in vitro* and *in silico* studies indicated that the compound possessed strong affinity for plasma proteins. Therefore, we suggest that JBC 1847 holds a promising potential as a novel topical agent for the clinical treatment of *S. aureus* skin infections, but pharmacokinetics and pharmacodynamics must be further studied.

## Data Availability Statement

The original contributions presented in the study are included in the article/Supplementary Material, further inquiries can be directed to the corresponding author/s.

## Ethics Statement

All animal procedure pertaining to this grant proposal to be carried out at Statens Serum Institut (SSI) are approved under a Danish Animal Experiment Expectorate. The Animal Facility at SSI is responsible for all the Animal Studies performed at SSI. The Animal facility follows the principles of GLP/GMP and has written SOPs for all procedures. The use and housing of the animals comply with the Danish legislation, which is based on the EU Directive 2010/63/EU on the protection of animals used for scientific purposes. The animals are housed in dedicated facilities and handled by experienced technicians. SSI does not have an official IACUC per se, but have an Animal Welfare Committee, which is the equivalent to IACUC. The committee gives guidelines for animal experimentations at SSI an arranges meetings to cover news related to 3 Rs and increase awareness of human use of experimental animals. All major procedures to be carried out at SSI as part of this application are already covered by Permissions by the Danish Animal Experiment Expectorate. As a general SSI procedure, The Animal Welfare Committee has decided that each individual experiment further has to be approved by the supervising laboratory animal veterinarians who are also part of the IACUC. This procedure has been put in pace to assure that each single experiment is in line with the permits allowances and to ensure a human use of animals with minimal suffering. These approvals are carried out on rolling basis. If additional procedures are to be included in the studies, this can be requested and amended to the permit on rolling basis. Case management of such an amendment at Danish Animal Experiment Expectorate would take a month of two.

## Author Contributions

TR, NJ, IH, SK, ES, SS, and JC: experimental work. TR and NJ: statistics, figures, and tables. TR, NJ, IH, SK, ES, AP, SS, JC, and RO: validation of results. TR, NJ, JC, and RO: wrote the manuscript. TR, AP, and RO: manuscript review. JC and RO: experimental design. All authors contributed to the article and approved the submitted version.

## Conflict of Interest

The authors declare that the research was conducted in the absence of any commercial or financial relationships that could be construed as a potential conflict of interest.
