# Supplementary material for: A Novel Promazine Derivative Shows High in vitro and in vivo Antimicrobial Activity Against Staphylococcus aureus
Source: Front Microbiol. 2020 Sep 30;11:560798. doi: 10.3389/fmicb.2020.560798 (PMC7555839; doi:10.3389/fmicb.2020.560798)
Supplement: Supplementary file 1 [file Data_Sheet_1.docx]

Supplementary Material

**Ames test**

**Positive controls**

| **Test Strain** | **±S9** | **Positive Control** | **Concentration** |
| --- | --- | --- | --- |
| TA98, TA100, TA1535 and TA1537 | + | 2-AA | 0.6 µg/well |
| WP2 uvrA (pKM101) | + | 2-AA | 4 µg/well |
| TA98 | - | 2-NF | 2 µg/well |
| TA100, TA1535 | - | SA | 0.24 µg/well |
| A1537 | - | CR-191 | 0.2 µg/well |
| WP2 *uvr*A (pKM101) | - | MNNG | 1 µg/well |

| **Chemical names of positive Control** | **CAS Number (Sigma-Aldrich)** |
| --- | --- |
| 2-Aminoanthracene (2-AA) | 613-13-8 |
| 2-Nitrofluorene (2-NF) | 607-57-8 |
| [Sodium azide](https://www.sigmaaldrich.com/catalog/substance/sodiumazide65012662822811) (SA) | 26628-22-8 |
| Acridine Mutagen (ICR 191) | 17070-45-0 |
| 1-Methyl-3-nitro-1-nitrosoguanidine (MNNG) | 70-25-7 |

**Ames Test procedure**

1) Tester strain cultures were grown overnight in nutrient broth at 37°C with shaking at 160 rpm to a density of 1-2x10^9^ colony forming units/ml (OD650~1.6). The S9 mix was prepared immediately prior to its use.

2) Top agar was prepared by adding 0.6% (w / v) agar powder and 0.5% (w / v) NaCl to water. Before use, 10 mL of a 0.5 mM histidine / biotin solution or 5 mL of a 0.5 mM tryptophan solution was added to each 100 mL of the melted top agar. Aliquot 1.6 mL of selective top agar supplemented with histidine/tryptophan was added into each glass tube.

3) 80 μL of negative control, test article preparation or positive control, 400 μL of S9 mixture or PBS buffer, and 80 μL of overnight culture of test strain was added to 1.6 mL of fused selective top agar (maintained at 45 ± 2 °C). It was mixed immediately and 540 μL of each well was added to a six-well plate (34.8 mm diameter) containing 5 mL of minimum glucose agar (1.5% agar, 2% glucose, VBE solution). Each treatment group was set up in three parallel (3 wells) and the vehicle / negative control was set up in six replicates.

The coagulated six-well plate was inverted and incubated in a 37±2°C incubator for 48 to 72 hours. Bacteria count was performed immediately after incubation.

**Acceptance criteria**

1) Positive control value: The average number of mutated colonies in the positive control group of the test strain was at least three times the average number of mutated colonies in the parallel negative control group to demonstrate the inherent sensitivity of the test strain to mutants and the integrity of the S9 mixture.

2) Positive result: To determine a test result positive, it must induce a concentration-dependent increase in the number of reverse mutant colonies or one or more increase in repeatability was observed on at least one strain (with or without the S9 metabolic activation system).

3) Negative result: If the test results do not meet the above two criteria, it is judged as negative

4) Suspicious positive result: Data with a biologically relevant increase that partially meet the positive criteria will be judged as suspected positive.

5) The average number of reverse mutated colonies of all test strains in the negative control and the positive control group should be comparable to historical laboratory data.

**Reagents and consumables**

Phosphate buffered saline, pH 7.4 (Sigma-Aldrich, USA; Cat #P4417)

Acetonitrile Chromasolv, gradient grade, for HPLC, ≥99.9% (Sigma-Aldrich, USA; Cat #34851) Ondansetron base powder (Enamine, Ukraine, Cat # EN300-117273)

Costar 96 Well Assay Blocks (Corning, USA; Cat # 3958)

MultiScreen HTS 96 Well Filter Plates (Millipore, Ireland; Cat # MSGVS2210)

UV-Star® 96 Well Microplate (Greiner Bio-One, Germany; Cat #655801)

Matrix Disposable pipette tips (ThermoScientific, USA; Cat ## 8041, 7622, 7321)

Flex-Tubes Microcentrifuge Tubes, 1.5ml (Eppendorf, Germany; Cat # 22364111)

Matrix Storage tubes, 1.4 ml (ThermoScientific, USA; Cat # 4247)

DMSO (Sigma-Aldrich, USA; Cat # 34869)

DMSO stock solution of the tested compound(s) 33.75 mM

Magnesium sulfate MgSO_4_*H_2_O (Fluka 83266)

Citric acid monohydrate (Enamine)

Potassium phosphate disbasic K2HPO4 (Helicon Am-0348)

Sodium ammonium phosphate Na_2_NH_2_PO_4_ (Sigma S9506)]

D-glucose monohydrate (Sigma 49158)

Agar (Sigma A1296)

Yeast extract (Bio Basic G0961)

Tryptone powder (Bio Basic TG217)

L-histidine (Sigma H6034)

Biotin (Sigma B4639)

L-tryptophan (Sigma T8941) Nutrient broth #2 (Oxoid CV0067)

Ampicillin (A9393)

2-Aminoantracene (Aldrich A38800)

2-Nitrofluorene (Sigma N16754)

4-Nitroquinoline N-oxide (Sigma N8141)

9-Aminoacridine (Enamine T5111202)

Sodium azide (Helicon Am-0639)

Beta-nicotinamide adenine dinucleotide 2`-phasphate reducted tetrasodium salt hydrate (NADPH) (Sigma N1630)

D-Glucose 6-phosphate sodium salt (Sigma G7879)

Sodium chloride (Sigma S3014)

Magnesium chloride MgCl_2_*6H_2_O (Sigma M2670)

Potassium chloride KCl (Sigma 60128)

Sodium phosphate dibasic Na_2_HPO_4_ (BioBasic S0404)

Sodium phosphate monobasic NaH_2_PO_4_*2H_2_O (BioBasic SB0879)

β-Naphtoflavone/sodium phenobarbital-induced rat liver S9 fraction, prepared from adult Wistar rats (Enamine)

35 mm Petri dish (Corning 430588)

1.5 ml Eppendorf tubes (Greiner Bio-one 616201)

96-well polypropylene plate (Nunc 249944)

384-well white plate (Corning 3574 or ThermoFisher Scientific 164564)

**Media**

*Vogel-Bonner E medium*: 1.4 mM MgSO_4_. H_2_O, 10 mM citric acid monohydrate, 57 mM K_2_HPO_4_, 17 mM Na_2_NH_2_PO_4_

*GM medium (glucose minimal agar medium)*: Vogel-Bonner E medium supplemented with 0.5% glucose, 1.5% agar

*GM liquid medium (glucose minimal medium)*: Vogel-Bonner E medium supplemented with 0.5% glucose

*Top agar*: 0.6% agar, 0.6% NaCl, 0.05 mM histidine and 0.05 mM biotin (for *S.typhimurium*) or 0.05 mM tryptophan (for *E.coli*).
